# Infiltration of innate and adoptive lymphoid cells in 4T1 and MC4-L2 breast cancer models

**DOI:** 10.22038/ijbms.2024.80535.17434

**Published:** 2025

**Authors:** Reihane Rasooli Tehrani, Hossein Asgarian-Omran, Saeid Taghiloo, Reza Valadan, Soheil Azizi, Abolghasem Ajami

**Affiliations:** 1 Department of Medical Immunology, School of Medicine, Mazandaran University of Medical Sciences, Sari, Iran; 2 Gastrointestinal Cancer Research Center, Mazandaran University of Medical Sciences, Sari, Iran; 3 Molecular and Cell Biology Research Center, Mazandaran University of Medical Sciences, Sari, Iran; 4 Department of Laboratory Sciences, Faculty of Paramedicine, Mazandaran University of Medical Sciences, Sari, Iran

**Keywords:** 4T1, Breast cancer, CD4^ +^ T-lymphocytes, CD8^ +^ T-lymphocytes, Innate lymphoid cells, MC4-L2

## Abstract

**Objective(s):**

Innate lymphoid cells (ILCs) are tissue-resident lymphocytes that have vital roles in activating further immune responses. However, due to their tumor-induced diversity, we decided to examine ILCs, T cells, and the associated cytokines in mouse models of breast cancer.

**Materials and Methods:**

4T1 and MC4-L2 cells were used to induce triple-negative and hormone-receptor-positive breast cancer, respectively. Tumor tissue was resected at early and late stages of tumor growth and used for further analysis. Total RNA was extracted and used in Real-Time PCR to analyze the expression of IFN-γ, IL-4, IL-10, IL-13, and IL-22. Tumor tissue was digested and used in a flow cytometric assay. H&E staining was used to examine the pathology of tumor progression.

**Results:**

Both tumor models showed a notable increase in T-cell frequency at the early stage of tumor growth. However, as the tumors progressed, the frequency of T cells significantly decreased, while the ILC component exhibited a significant increase in tumor progression. Gene analysis indicated a significant increase in the inflammatory to anti-inflammatory cytokine ratio during tumor progression in the tumor model. In contrast, this ratio was considerably reduced in advanced MC4-L2 tumors. Both tumor models showed the development of invasive breast carcinoma and lung metastasis in advanced tumors.

**Conclusion:**

Our study highlighted the expansion of ILCs during tumor progression in two distinct breast cancer models with different immunogenicity. These findings suggest that ILCs may actively modulate the tumor microenvironment during the advanced stage of tumor growth.

## Introduction

According to the latest global statistics in 2020, breast cancer is the most prevalent cancer worldwide (1). Metastasis causes a decrease in the 5-year survival rate from 80% to 25% (2). Depending on the hormone receptor expression (estrogen receptor (ER), progesterone receptor (PR), human epidermal growth factor receptor 2 (HER2)) and histological features, breast cancer can be divided into luminal A-like (ER^+^, PR^+^, HER2^-^), luminal B-like HER2^-^ (ER^+^, PR^+/-^, HER2^-^), luminal B-like HER2^+^ (ER^+^, PR^+/-^, HER2^+^), HER2-enriched (ER^-^, PR^-^, HER2^+^), and triple-negative breast cancer (TNBC) (ER-, PR-, HER2-) (3). Among different breast cancer subtypes, TNBC is more immunogenic. It has a higher mutational load, TIL infiltration, and PD-L1 expression (4-6). Target therapy in breast cancer includes anti-PD-L1 (atezolizumab) and anti-PD-1 (pembrolizumab) in combination with nab-paclitaxel or chemotherapy for metastatic TNBC. However, immune checkpoint inhibitors (ICI) have low therapeutic efficacy in TNBC patients (7-9). ICI therapy focuses mainly on T cells, while the innate immune cells are also present in the tumor microenvironment. In the context of innate immunity, Innate lymphoid cells (ILCs) are comparable to T cells, distributed in barrier and non-barrier tissues that enable them to respond rapidly to tissue remodeling (10). 

ILCs are a group of immune cells with the morphology of lymphoid cells. They colonize lymphoid and barrier tissues during fetal development and have transcriptional and functional similarities to both cytotoxic and helper T cells. However, ILCs lack somatic recombination, antigen-specific receptors, and lineage-specific markers (11, 12). Moreover, they have varied abilities to circulate, expand, and renew, making them the innate counterparts of T cells (13). They likely appeared later in evolution than T and B lymphocytes, as suggested by phylogenetic studies (14). The ILCs family is divided into five subclasses that are approved by the International Union of Immunological Societies: NK cells and ILC1, both depend on transcription factors T-bet and produce interferon-γ (IFN-γ) and the tumor necrosis factor (TNF-α), but NK cells also depend on transcription factor Eomes; ILC2 with specific transcription factor GATA3 and the production of interleukin (IL)-4, IL-5, IL-9, IL-13, and amphiregulin (AREG); ILC3 depend on RORγt as transcription factor and produce IL-17, IL-22, granulocyte-macrophage colony-stimulating factor (GM-CSF) and LTi cells (15).

Regarding cancer immunity, initial reports have shown that ILCs exist in lymphoid structures in human colon, lung, and breast tumors and express activating molecules, including MHC-ІІ (16-20). Some anti-tumor or tumor-promoting functions have been attributed to ILCs. NCR^+^ ILC3 cells may contribute to forming protective tumor-associated tertiary lymphoid structures (16). ILC2 via IL-13 production can support the activation of monocytic myeloid-derived suppressor cells (M-MDSCs) (21). Conversely, it has been reported that invigorating tumor ILC2 promoted tissue-specific CD8+ T cells and amplified responses to anti-PD-1 (22). Despite research about ILCs function, the link between ILCs and T-cell infiltration in tumors with different immunogenicities has not been explored. To address this question, we used two distinct breast cancer models. We used syngeneic models of TNBC and HR^+^ by the use of 4T1 and MC4-L2 cell lines in BALB/c mice. Immune cell profiling and cytokine gene analysis were performed in the early and advanced stages of orthotopic breast carcinoma progression.

## Materials and Methods

***Cell lines***

4T1 and MC4-L2 are mouse mammary carcinoma cell lines derived from BALB/c mice. The 4T1 mammary tumor cells exhibit aggressive metastatic characteristics similar to human TNBC (23). In contrast, the MC4-L2 cell line induces metastatic mammary tumors with high expression of ER, PR, and HER2 (24). The 4T1 cell line was a gift from Dr. Abedian-kenari, and the MC4-L2 cell line was purchased from the Iranian Biological Resource Center (IBRC) (Iran). 4T1 cells were maintained in the RPMI-1640 medium (Gibco, Germany), while MC4-L2 cells were cultured in the DMEM-F12 (Biowest, Korea) medium. Each culture medium was supplemented with 10% heat-inactivated fetal bovine serum (Anacelltech, Iran), 100 units/ml of penicillin, and 100 μg/ml of streptomycin (Biosera, Korea). Cell lines were maintained in a 37 °C incubator with 5% CO_2_ and 90% humidity. 

***CFSE cell proliferation analysis***

Cultured 4T1 and MC4-L2 cells in the logarithmic phase of growth were harvested and stained with 5-(and 6)-carboxyfluorescein diacetate succinimidyl ester of CFDA SE (CFSE) (Biolegend, USA) according to the manufacturer’s protocol. Cells were then seeded in 6-well plates at 130×10^3 ^cells/well. The amount of absorbed stain was analyzed by a PAS flow cytometer system (Partec GmBH, Germany) every day, starting from day 0 (the day that staining was performed) until three days later. Mean Fluorescent Intensity was calculated by FlowJo software v10.8.1, and a related histogram was plotted.

***In vivo studies***

Female BALB/c mice between six and eight weeks of age were obtained from the Razi Vaccine and Serum Research Institute (Iran). We had three groups of mice. To establish tumor models, 1×10^6 ^4T1 and 1×10^6^ MC4-L2 cells in 100 µl PBS were orthotopically implanted in the right 4th mammary gland. Additionally, 100 µl PBS (1x) was injected into the mammary gland of mice used as a normal group. Tumor size was measured using a caliper, and tumor volume was calculated. Tumor volume (mm^3^) = ((length × width^2^)/2). All experimental studies for both mammary tumor models were conducted at two time points based on tumor size. The first time point was when tumor volume reached 25–50 mm^3^, referred to as early tumors. The second time point was when tumor volume reached 250–400 mm^3^, referred to as advanced tumors. Additionally, we used a normal group as a control in all studies. *In vivo* study protocols were approved by the Ethical Committee of Mazandaran University of Medical Sciences (IR.MAZUMS.REC.1399.795).

***Flow cytometry***

Tumor specimens and normal mammary tissue from the normal group were isolated at indicated tumor volumes. Tissues were dissected into smaller fragments using scalpels and then suspended in an enzymatic solution containing 1 ml collagenase A (0.5 mg/ml) (Roche, Switzerland), 1 ml hyaluronidase (1 mg/ml) (Sigma-Aldrich, USA), and 1 ml complete cell culture medium, placed in a 37 °C shaker incubator for 2 hr (This ratio of enzyme solution was used for tissue samples up to 40 mg in size). Digested tumors were filtered through a 70 μm cell strainer (Sorfa, China) and washed twice with PBS in order to eliminate the enzymatic solution. Finally, the isolated cells from each specimen were individually stained following anti-mouse antibodies including anti-CD45 (30-F11, PE/Cyanine7), anti-CD3ε (KT3.1.1, APC), anti-CD4 (GK1.5, PE), anti-CD8α (QA17A07, FITC), anti-CD117 (c-Kit) (2BB, PE), anti-IL-33Rα (ST2) (DIH4, APC), and Lineage Cocktail-FITC (anti-mouse CD3ε, clone 145-2C11; anti-mouse Ly-6G/Ly-6C, clone RB6-8C5; anti-mouse CD11b, clone M1/70; anti-mouse CD45R/B220, clone RA3-6B2; anti-mouse TER-119/Erythroid cells, clone Ter-119) (Biolegend, San Diego, California, USA), for 45 min at 4 °C followed by washing with PBS with 0.5 % BSA buffer and then all samples were fixed with 1% formaldehyde. Samples were run on a BD FACS Calibur flow cytometer (BD Biosciences, USA), and acquired data were analyzed using FlowJo software v10.8.1. Total ILCs were gated as lineage-negative and CD45-positive cells; then, by the use of CRTH2 and c-Kit markers, cells were divided into group 1 ILC (ST2^−^ c-Kit^−^), group 2 ILC (ST2^+^ c- Kit ^+/−^), and group 3 ILC (ST2^−^ c-Kit^+^). In addition, T cells gated as CD45+ and CD3+ cells are further divided into CD4+ (T helper) and CD8+ (T cytotoxic) cells.

To limit flow cytometric errors, we used three types of controls. To eliminate the auto fluorescent of cells, we used a negative control. Isotype-matched control antibodies were also used to eliminate background staining caused by an antibody’s non-specific binding. Compensation controls, including single-stained samples for each antibody, were used to limit the spillover of each fluorophore into the other detectors.

***RNA isolation and cDNA synthesis***

Total RNA was extracted using the AccuZol ^TM^ Total RNA Extraction Reagent (Bioneer, South Korea) based on the manufacturer’s instructions. The purity and concentration of RNA were determined using a nano-spectrophotometer (WPA, England) and agarose (YTA. Iran) gel electrophoresis. Total RNA (2 µg/µl) was applied for complementary DNA (cDNA) synthesis using the cDNA Synthesis Kit (YTA, Iran) according to the manufacturer’s instructions.

***Quantitative real-time PCR***

Real-time PCR was performed using the Step One plus ABI system (Applied Biosystems, USA) using SYBR green detection dye (Ampliqon, Denmark). Primer sequences used for qPCR are listed in Table 1. Primer efficiencies were calculated using the Lin-reg PCR application (v 2017.1.0.0). Glyceraldehyde-3-phosphate dehydrogenase (GAPDH) was used as an internal control to normalize gene expression. The PCR thermal cycling conditions were as follows: initial denaturation at 95 °C for 15 min, followed by 40 cycles of 95 °C for 30 sec, 60 °C for 30 sec, and 72 °C for 30 sec. Finally, the relative expression level of each molecule was calculated using the Pfaffl method.

***Histology***

Tissues were isolated, fixed in 10% buffered formaldehyde at room temperature (RT), and then embedded in paraffin. Using a Rotary Leica microtome (Leica Biosystems, Germany), 5 μm sections were cut. The sections were deparaffinized using xylene, rehydrated with ethanol and then washed with distilled water. Subsequently, the sections were stained with hematoxylin and eosin (H&E). Sections were dehydrated through graded ethanol and cleared using xylol (25). Eventually, sections were prepared and mounted with a cover glass for further analysis.

***Statistical analysis***

Statistical analyses were performed using GraphPad Prism 8. Quantitative data are represented as mean ± SEM. Analysis was conducted using the Shapiro-Wilk test to determine the normality distribution of the obtained data. For comparison between two groups, the t-test was employed. For comparisons involving more than two groups, one-way analysis of variance (ANOVA) followed by Tukey’s test was used. *P*<0.05 was considered statistically significant.

## Results

***Differential tumor growth kinetics in 4T1 and MC4-L2 orthotopic breast cancer models***

**Figure 1:**
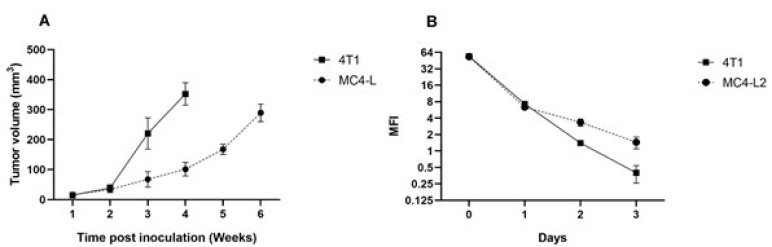
Tumor burden of 4T1 and MC4-L2 murine breast carcinoma models

To gain a better insight into the immune system’s interactions in breast cancer, we selected two distinct murine mammary cancer cell lines, 4T1 and MC4-L2, representing different pathological types. Following tumor challenge, 4T1 cells established primary tumors within 7 and 10 days post injection (p.i), while it took 14 to 16 days p.i for MC4-L2 tumor cells to induce palpable tumors. Measurements of the primary tumor volume in Figure 1A revealed that the 4T1 model exhibited more aggressive tumor growth and faster lung metastasis compared to the MC4-L2 model. In addition, *in vitro* CFSE analysis revealed that the 4T1 cell line has a higher proliferation rate than the MC4-L2 cell line, as indicated in Figure 1B. 

***Dynamic of ILCs subset infiltration during tumor progression in 4T1 and MC4-L2 breast tumor models***

**Figure 2:**
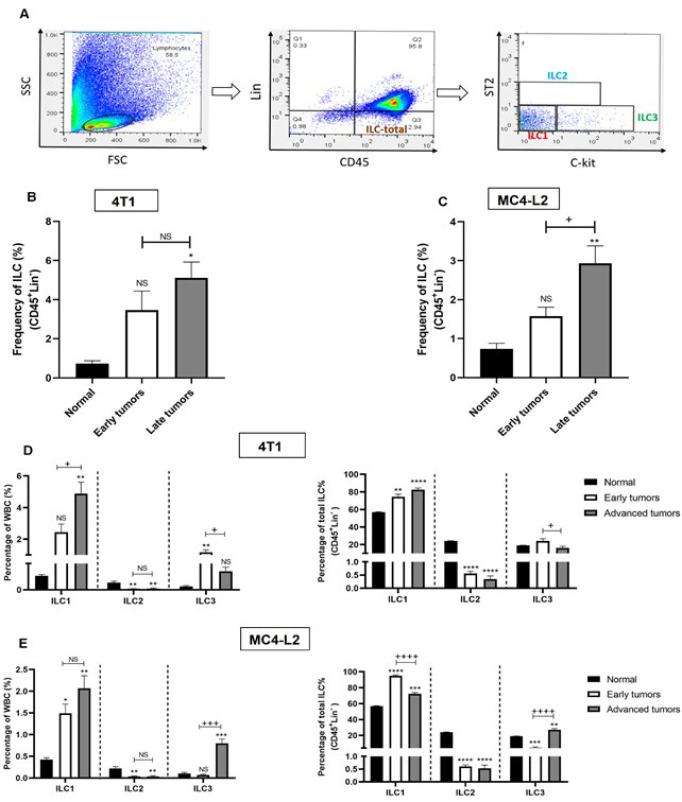
Changes in ILC infiltration over the course of tumor development in mice

We examined the frequency and subset distribution of ILCs in tumor tissue when the tumor reached the specific tumor volume indicated in Figure 1A. Measurement of the ILCs population in normal mammary tissue revealed that ILCs comprised less than 1% of total immune cells. Implantation of 4T1 and MC4-L2 breast cancer cells induced a profound accumulation of ILCs within tumors over time. By late stages, total tumor-associated ILC levels had increased over fivefold in 4T1 and threefold in MC4-L2, compared to normal tissue (Figure 2A-C). In Early 4T1 tumors, ILC3 percentages rose noticeably (*P*=0.0013) among infiltrating leukocytes with no change in ILC1 frequency (*P*=0.13). After tumor progression, ILC3 levels returned to baseline, whereas ILC1 levels noticeably increased (to 4.8%) (*P*=0.0021). Unlike ILC1 and ILC3, the frequencies of ILC2 were even lower than normal (*P*=0.0094) and remained constant. To investigate the distribution of ILCs subsets among total ILCs, we observed that in normal tissue, ILC1 predominated at 57%, with ILC2 and ILC3 representing 24% and 19%, respectively. In 4T1 tumors, similar to normal tissue, ILC1 was the predominant subtype, with its proportions amplifying to 75% of total ILCs in early tumors and remaining elevated afterward. Meanwhile, ILC3 frequency declined sharply after tumor growth, and ILC2 was scarce in tumors compared to normal tissue (Figure 2D). Evaluation of ILC subset alteration among total tumor-infiltrating white blood cells (WBC) in the MC4-L2 model showed that early in response to the tumor, only the ILC1 percentage was altered and increased (*P*=0.026). Later, ILC3 frequency significantly rose (*P*=0.0005), in addition to a slight increase in ILC1 (*P*=0.2). Similar to the 4T1 tumors, analysis of ILCs subsets among total ILCs in the MC4-L2 model revealed that ILC1 comprised the most prevalent subset (approximately 95% of total ILCs), followed by ILC3 (4.4%) and ILC2 (less than 1%). An inverse relationship between ILC1 and ILC3 was observed in MC4-L2 tumors,, with ILC1 increasing and ILC3 decreasing in early tumors, then reversing with tumor progression (Figure 2E).

***Kinetics of CD4*** ^+^ ***and CD8*** ^+^ ***T cell infiltration in 4T1 and MC4-L2 breast tumor models***

**Figure 3:**
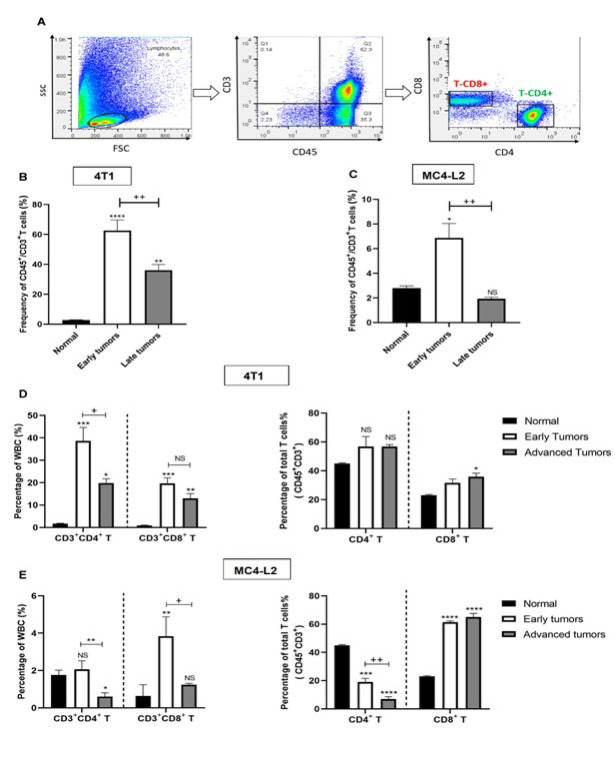
Changes of CD4+ to CD8^+^ T cells over the course of tumor development in mice

T lymphocytes play an essential role in cancer immunosurveillance. Therefore, we evaluated the kinetics of T cells in mammary carcinoma models during different stages of tumor growth when tumors reached the specified volumes (Figure 1A). Based on the pan-immune cell marker CD3, T cells made up 2.8% of WBC in normal mammary tissue. In the 4T1 tumor model, the total T cell population increased significantly to 62.65% early after tumor challenge (*P*<0.0001) but then declined to 36.08% (*P*=0.0082) as the tumor progressed (Figure 3A and B). In detailed analysis, initially, both CD4^+^ (up to 38.6%) and CD8^+^ (up to 19.7%) T cell subsets among WBC significantly increased compared to normal levels (*P*=0.0003 and *P*=0.0007, respectively), with CD4^+^ T cells dominating. Later, CD4^+^ T cells considerably declined to 19.8% (*P*=0.012), while CD8^+^ T cells remained similar. In the next step, we also analyzed the frequency of CD4^+^ and CD8^+^ subsets as a proportion of CD3^+^ T cells. In normal mammary tissue, the T cell distribution consists of 45% CD4^+^ cells and 23% CD8^+^ cells in a 2:1 ratio. After tumor challenge in 4T1 model, also CD4^+^ cells were the dominant subtype, comprising around 56%, while in advanced tumors, CD8^+^ T cells rose to 35.86% (*P*=0.012), caused a reduction in CD4/CD8 ratio from 2:1 to 1.58:1 (Figure 3D). 

The MC4-L2 model showed a similar trend in total T cell frequency as in the 4T1 tumor model but with a different frequency. Total T cells increased to about 6.87 at the early stage (*P*=0.013) but later significantly declined to near normal levels (1.93%) (*P*=0.0054) (Figure 3C). Only CD8^+^ T cells in total WBC increased (*P*=0.0077), while CD4^+^ T cells remained constant in 4T1 tumors. Later in the advanced stage, both subsets declined to normal levels (*P*=0.013 and *P*=0.003). The distribution of CD4^+^ and CD8^+^ cells as a proportion of the total T cell population was modulated in MC4-L2 models. In contrast to normal tissue and 4T1 tumors, CD8^+^ T cells comprised the majority (60.14%) of total T cells in both early and late tumors (*P*=0.0001), exceeding CD4^+^ T cells, which comprised only 19% in early tumors and declined to 6.8% in late-stage tumors (*P*=0.0086) (Figure 3E).

***Analysis of cytokine profiles of tumor microenvironment***

**Figure 4:**
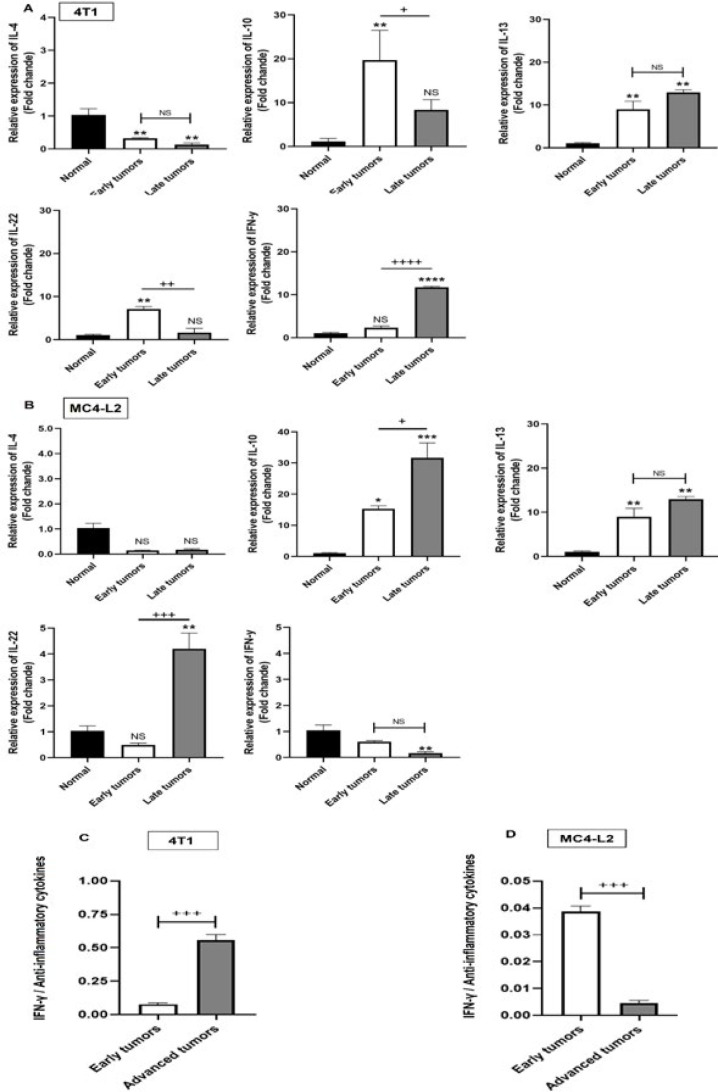
Relative mRNA expression of IL-4, IL-10, IL-13, IL-22 and IFN-γ in murine model of breast cancer

We employed local cytokine expression profiling to reveal the connection between immune cell infiltration and their activation in the tumor microenvironment. We examined cytokines associated with each ILC and T cell subset, including IFN-γ, IL-4, IL-10, IL-13, and IL-22. IFN-γ is a pro-inflammatory cytokine that can be produced by ILC1, Th1, and CD8^+^ T cells. Conversely, IL-4, IL-10, and IL-13 are anti-inflammatory type 2 cytokines produced by ILC2 and Th2 cells. IL-22 is released by Th22 and ILC3 as part of Type 3 immunity. In 4T1 tumors, significant elevations in IL-10 (*P*=0.003), IL-13 (*P*=0.006), and IL-22 (*P*=0.001) were detectable at early stages. During late carcinogenesis, IL-10 (0.036) and IL-22 (*P*=0.003) declined to baseline; conversely, IFN-γ markedly up-regulated (*P*<0.0001) (Figure 4A). In early MC4-L2 lesions, only a remarkable rise in IL-10 gene expression was observed compared to normal mammary tissue (0.026). Advanced-stage MC4-L2 tumors demonstrated further up-regulation of IL-10 (*P*=0.014), along with significant elevations in IL-13 (*P*=0.0001) and IL-22 (0.002). Paradoxically, IFN-γ expression decreased even further than the normal level (*P*=0.006) (Figure 4B). In both tumor models, IL-4 was down-regulated compared to normal breast tissue. Evaluating the equilibrium between IFN-γ and anti-inflammatory cytokines (IL-4 + IL-10 + IL-13) revealed tumor progression is accompanied by an increased inflammatory balance in 4T1 tumors (*P*=0.0004), in contrast to MC4-L2 tumors (Figure 4C, D).

***H*** ***&*** ***E histology analysis***

**Figure 5:**
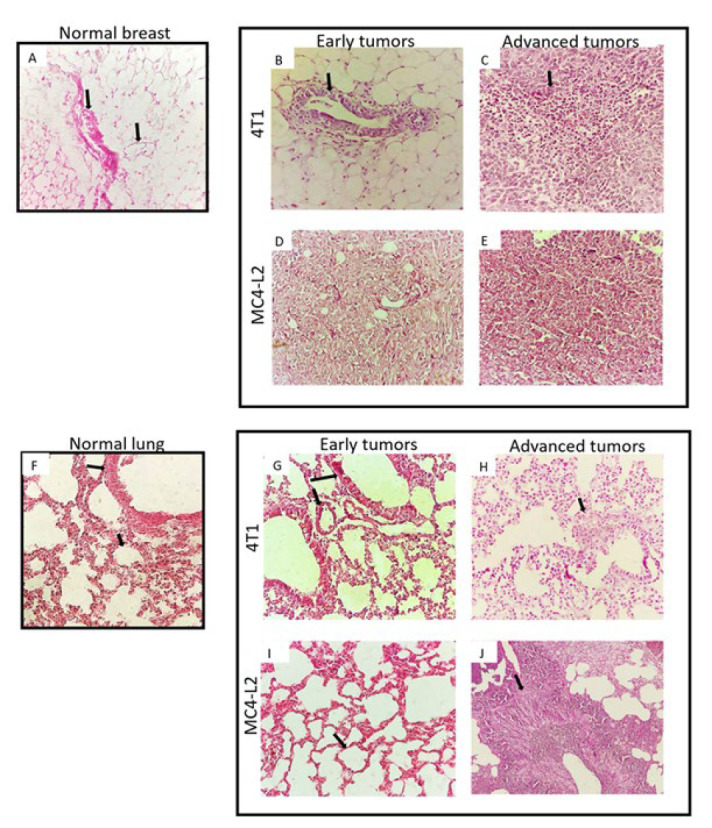
Normal histology and histopathology of breast and lung of control and tumor groups of mice

Histological examination using hematoxylin and eosin staining was conducted to investigate tissue remodeling and distant metastasis in mice. The primary tumors and lungs of individual mice were analyzed. In early tumors with an average volume of 25–50 mm^3^, the 4T1 mammary carcinoma showed tumor cells confined to the ductal structures within the mammary gland, leading to a diagnosis of ductal carcinoma *in situ* (DCIS). On the other hand, in the MC4-L2 model with a similar average tumor volume, tumor cells were distributed throughout the mammary gland, resulting in a diagnosis of invasive carcinoma (IC). At this early stage, there was no evidence of tumor cell migration or lung metastasis in mice from either model. However, when the tumors reached a volume of 250–400 mm^3^, IC occurred in the 4T1 model, and lung metastasis was confirmed in both the 4T1 and MC4-L2 tumor-bearing mice (Figure 5).

**Table 1:** Specific primer sequences used for murine gene expression

Gene	Directions and sequence (5’-3’)	Product Size (bp)
IL-4	F: TAGTTGTCATCCTGCTCTTC	173 bp
	R: CTCTGTGGTGTTCTTCGT	
		
IL-10	F: GTGAAGACTTTCTTTCAAACAAA	108 bp
	R: CATTTCCGATAAGGCTTGG	
		
IL-13	F: CTCTCCCTCTGACCCTTA	98 bp
	R: GTCCACACTCCATACCAT	
		
IL-22	F: GACCAGAACATCCAGAAGAAT	121 bp
	R: AAGCATTTCTCAGAGACATAAAC	
		
IFN-γ	F: ATTACTACCTTCTTCAGCAACAG	176 bp
	R: AATCAGCAGCGACTCCTT	
		
GAPDH	F: GGAGAAACCTGCCAAGTATGA	90 bp
	R: TCCTCAGTGTAGCCCAAGA	


A) Tumor growth curve. 4T1 (1×10^6^ cells/mouse) and MC4-L2 (1×10^6^ cells/mouse) were implanted into the mammary fat pad of female BALB/c mice (n = 4). Marked points indicate tumor collection size for further analysis (25–50 mm^3^ early tumors, 250–400 mm^3^ advanced tumors). B) CFSE cell proliferation analysis is represented as mean fluorescence intensity (MFI).

Flow cytometry analysis of innate lymphoid cells (ILCs) from primary tumors when reached indicated sizes in Figure 1 and normal mammary gland. (A) Representative gating strategy for ILCs subsets identification. Frequencies of total ILCs identified as CD45+ Lin− cells out of lymphocytes in 4T1 (B) and MC4-L2 tumors (C). Proportion of ILC1 (c-Kit– ST2-), ILC2 (c-Kit +/- ST2+), and ILC3 (c-Kit+ ST2-) as a percentage of total ILCs (Lin- CD45+ cells) or as a percentage of total WBC (CD45+ cells) in 4T1 (D) and MC4-L2 (E) tumors. Data are shown as mean ± SEM from four mice in each group. One-way ANOVA with Tukey’s multiple comparison test was used for statistical analysis. Statistical significance is indicated as NS (not significant). (*) represents the comparison of each stage with the normal group, * *P*<0.05, ** *P*<0.01, *** *P*<0.001, **** *P*<0.0001. (+) represents the comparison between the early and advanced stages, + *P*<0.05, +++ *P*<0.001, ++++ *P*<0.0001.

Flow cytometry analysis of individual T cell populations from primary tumors when reached indicated sizes in Figure 1 and normal mammary gland. (A) Representative gating strategy. Frequency of CD3^+^ T cells out of CD45+ cells in 4T1 (B) and MC4-L2 tumors (C). Proportion of CD4+ and CD8+ T cells were calculated based on their frequency in total T cells (CD3^+^ cells) and also total WBC (CD45+ cells) in 4T1 (D) and MC4-L2 tumors (E). Data are shown as mean ± SEM from four mice in each group. One-way ANOVA with Tukey’s multiple comparison test was used for statistical analysis. Statistical significance is indicated as NS (not significant). (*) represents the comparison of each stage with the normal group, * *P*<0.05, ** *P*<0.01, *** *P*<0.001, **** *P*<0.0001. (+) represents the comparison between the early and advanced stages, + *P*<0.05, +++ *P*<0.001, ++++ *P*<0.0001.

Real-time quantitative PCR was used to quantify IL-4, IL-10, IL-13, 1L-22, and IFN-γ mRNA expression level in 4T1 (A) and MC4-L2 tumors (B) and normal group relative to housekeeping gene (GAPDH). Ratio of IFN-γ to anti-inflammatory cytokines (IL-4 + IL-10 + IL13) in 4T1 (C) and MC4-L2 tumors (D). Quantitative results are expressed as the mean ± SEM (n = 4). One-way ANOVA with Tukey’s multiple comparison test was used for statistical analysis (A and B). Unpaired T-test was used for statistical analysis (C and D). Statistical significance is indicated as NS (not significant). (*) represents the comparison of each stage with the normal group, * *P*<0.05, ** *P*<0.01, *** *P*<0.001, **** *P*<0.0001. (+) represents the comparison between the early and advanced stages, + *P*<0.05, ++ <0.01, +++ *P*<0.001, ++++ *P*<0.0001.

Hematoxylin and Eosin (H&E) staining, original magnification 400X. (A) Normal breast tissue with blood vessel and duct. (B) Early 4T1 tumors with tumor cells in duct structure. (C) Advanced 4T1 tumor with highly proliferative tumor cells and necrotic center. (D and E) Early and advanced MC4-L2 tumors with distributed tumors cells. Alveolus and bronchiole in normal (F), early 4T1 (G), and early MC4-L2 (I) lung tissue. Metastatic cells in advanced 4T1 (H) and advanced MC4-L2 (J) lung specimens.

## Discussion

In this study, we examined the changes of ILC1, ILC2, and ILC3 frequency in innate immunity, CD4^+^ and CD8^+ ^T cells in adoptive immunity, and the expression of their associated cytokines (IFN-γ, IL-4, IL-10, IL-13, and IL-22) in 4T1 and MC4-L2 breast tumor models. Total ILCs increased with tumor progression in both cancer models (4T1 and MC4-L2). The expansion of the ILC population could be mediated by migration from circulation to specific sites due to the expression of chemokine receptors (26) and adhesion molecules (27, 28) or *in situ* ILC proliferation (29). Studies in the MMTV-PyMT (PyMT) breast tumor model revealed that ILC1 represents immunosurveillance outcomes in breast tumors. Cytotoxic ILC1, with the ability to produce granzyme B, increased in the tumor site. Notably, these cells were distinct from NK cells and were dependent on IL-15 derived from cancer cells. Furthermore, using the TCGA database, the ILC1 mediated immunity was also detected in humans (30, 31). 

We observed that ILC1 was the most abundant subtype in both early 4T1 and MC4-L2 models. These data may indicate that ILC1 is also involved in the initial immunosurveillance against 4T1 and MC4-L2 models. After tumor progression, there was a small increase in the ILC1 compartment in the 4T1 model, accompanied by IFN-γ gene up-regulation. This might indicate that ILC1 has a protective role even in the late stage of 4T1 tumor growth. In contrast, in advanced MC4-L2 tumors, ILC1 frequency decreased, possibly due to a mechanism of tumor escape. The modulation of ILC1 in the advanced stage was accompanied by an increase in ILC3 frequency in MC4-L2, in contrast to a decrease in ILC3 in the 4T1 tumor. This subtype alteration could be explained by the plasticity between ILC1 and ILC3, which is controlled by the cytokine gradient of the tumor microenvironment (32). In both 4T1 and MC4-L2 models compared to the normal group, ILC2 frequency significantly declined and had no alteration after tumor progression. It has been demonstrated that activated ILC2 increased in malignant human breast tissue more than in benign tissue. However, in this study, ILC1 and ILC3 were more abundant in tumor sites, but only ILC2 had a significant difference between benign and malignant tissue (18). In our experiment, the maximum level of ILC3 frequency was coupled with up-regulation of the IL-22 gene, which was detected in the early stage of 4T1 tumor growth and the late stage of MC4-L2 tumor development. Consistent with the tumor-promoting role of IL-22 (33, 34), ILC3 could have a tumor-promoting effect in both models. Our data is in line with previous findings, which showed an increase in ILC3 and IL-22 levels in 4T1 tumors. ILC3 was the primary cellular source of IL-22. They also suggested that IL-22 acts through EMT regulation (35). Another pro-tumor mechanism of ILC3 was found in 4T1 tumors. Recruitment of RORgt^+^ ILC3 mediated tumor stromal cells to release RANKL, which led to lymph node invasion. Patient cohort study also declared that in primary tumors, CCL21 up-regulation and RORgt^+^ ILC3 infiltration were correlated to enhanced draining lymph node metastasis in basal-like breast cancer but not in HER2+ or luminal A/B subtype (36).

In our study, tumor induction in 4T1 and MC4-L2 models led to increased CD3^+^ T cell population at the early stage, but their frequency declined during tumor progression. Of note, the frequency of CD3^+^ T cells was higher in the 4T1 model than in the MC4-L2 model, suggesting that the 4T1 model is more immunogenic. It has been announced that HR^+^ tumors have low levels of HLA-I expression, which could cause T cell and NK cell exclusion (37) and might be related to our observation of the low levels of T cells in the MC4-L2 model. In 4T1 tumors, CD4^+^ cells were the dominant subtype of T cells, and their frequency remained constant. Notably, a significantly higher percentage of CD8^+^ cells were observed in advanced cancer tissue than in normal tissue. The enhancement of cellular immunity in the 4T1 tumor model is also explained by other studies, in which tumor progression was associated with an increase in CD8^+^ Gzmb cells (38), CD8/CD4 equilibrium (39), and IFN-γ^+^ CD4 cells (40). The studies mentioned above reported different results regarding the total CD3^+^ T cells. Differences in the timing and size of tumors in each experiment are likely the reasons for this discrepancy. MC4-L2 tumors showed a higher abundance of CD8^+^ T cells than CD4^+^ cells, an adverse modulation of CD3^+^ T cell subsets compared to 4T1 tumors. Based on the very low total T cell frequency, it seems that a high percentage of CD8^+^ cells does not play a prominent role in the immune defense of MC4-L2 tumors.

In gene expression analysis, we observed an enrichment of IL-10, IL-13, and IL-22 in early 4T1 tumors, while IL-10 and IL-22 were down-regulated and IFN-γ was up-regulated in advanced tumors. An increase in the IFN-γ/ type 2 cytokine ratio could be due to the rise in ILC1 and CD8^+^ T cells in advanced tumors. These changes in the cytokine gene are consistent with the report by Taylor *et al*. They showed Macrophages (MQ) are primary cells recruited to the tumor site, followed by neutrophil and T cells, accompanied by a decrease in IL-4 and IL-10 and an increase in pro-inflammatory cytokines. Despite anti-tumor immune responses, 4T1 tumors have a myeloid-enriched microenvironment that promotes tumor progression and metastasis, as we have seen in late tumors (38, 41). Among the evaluated cytokines in the early stage of MC4-L2 tumors, only a rise in IL-10 expression was detected; after tumor progression, IL-13 and IL-22 were overexpressed in addition to IL-10. In contrast, IFN-γ was down-regulated even less than baseline. Based on the low proportion of T cells, especially CD4^+^ T, and the even further decline of this subtype with tumor progression, we can assume that the source of these cytokines are myeloid cells and ILCs present in the tumor microenvironment, as the ILC3 population and IL-22 expression increased at the same time. The lack of effector immune cells and the presence of myeloid cells could lead to continuous increase in anti-inflammatory cytokines in advanced MC4-L2 tumors. Regarding IFN-γ down-regulation, our observation is in accordance with the suppression of IFN-γ production by CD4^+^ cells in E0771 and PyMT breast cancer models. Which further limited classical activation and anti-tumor function of monocytes/macrophages recruited to the tumor and led to increased growth of primary and metastatic breast tumors (42). Despite the role of IL-4/IL-4R in breast cancer progression in human and mouse models, we detected down-regulation of IL-4 in both 4T1 and MC4-L2 tumors (43-45). Elevated IL-13 expression was a common feature of both studied models. Consistent with the role of IL-13 in the activation of MQ and MDSC, the up-regulation of IL-13 could be related to the defined role of MQ and MDSC in breast cancer progression (46-48). Due to limitations in this study, we did not assess cytokines at the protein level and examine the activation status and anti-tumor functions of ILCs and T cells *in vivo* and *in vitro* to declare whether these cells are active or exhausted. Further studies are needed to answer these questions.

## Conclusion

We illustrate that apart from a distinct inflammatory tumor microenvironment and T cell infiltration in 4T1 tumors compared to MC4-L2 tumors, the frequency of ILCs has remarkably increased in both advanced tumors. These results indicate the role of the ILC population in cancer immune response. Based on our observation of ILC increase in the breast cancer models and the role of tumor ILC2 in invigorating anti-PD-1 treatment of aggressive cold pancreatic (PDAC) model (22), it seems that these cells could be valuable targets for enhancing other treatments, especially in immune deficiency cancers like breast cancer.

## Declaration

We acknowledge the use of ChatGPT to improve the language and readability of the text.
